# Follow-up studies on the immune status of patients with Hodgkin's disease after splenectomy and treatment, in relapse and remission.

**DOI:** 10.1038/bjc.1977.199

**Published:** 1977-09

**Authors:** B. W. Hancock, L. Bruce, I. R. Dunsmore, A. M. Ward, J. Richmond

## Abstract

Sixty-two patients with Hodgkin's disease have been followed for one year from the start of treatment. Immunological assessments were repeated after intensive treatment, in patients relapsing and in those in remission at one year. In patients achieving remission, overall cellular immunity, after deteriorating with therapy, particularly cytotoxic chemotherapy, returned to pre-treatment levels in remission when there was little evidence of cellular immune disturbance. Serum IgG and IgM levels fell with intensive chemotherapy in splenectomized patients. IgA and IgM levels were lower (irrespective of splenectomy or therapy status) in remission than at presentation or after treatment. Relapse or non-response was usually associated with deteriorating cellular immunity. Herpes zoster/varicella and candida infections (seen in 6 patients) were preceded by, or associated with, deterioration of cellular immunity.


Br. J. Cancer (1977) 36, 347

### FOLLOW-UP STUDIES ON THE IMMUNE STATUS OF PATIENTS WITH HODGKIN'S DISEASE AFTER SPLENECTOMY AND TREATMENT, IN RELAPSE AND REMISSION

B. W. HANCOCK,* L. BRUCE,* I. R. DUNSMORE,† A. MILFORD WARD‡ AND J. RICHMOND*

*From the *Department of Medicine, Royal Hospital, the †Department of Probability and Statistics and the ‡Department of Immunology, Academic Division of Pathology, University of Sheffield*

Received 2 March 1976 Accepted 13 May 1977

**Summary.—Sixty-two patients with Hodgkin's disease have been followed for one year from the start of treatment. Immunological assessments were repeated after intensive treatment, in patients relapsing and in those in remission at one year. In patients achieving remission, overall cellular immunity, after deteriorating with therapy, particularly cytotoxic chemotherapy, returned to pre-treatment levels in remission when there was little evidence of cellular immune disturbance. Serum IgG and IgM levels fell with intensive chemotherapy in splenectomized patients. IgA and IgM levels were lower (irrespective of splenectomy or therapy status) in remission than at presentation or after treatment. Relapse or non-response was usually associated with deteriorating cellular immunity. Herpes zoster/varicella and candida infections (seen in 6 patients) were preceded by, or associated with, deterioration of cellular immunity.**

DISTURBANCES in immunity may be seen in patients with generalized Hodgkin's disease (Young *et al.*, 1972; Hancock *et al.*, 1977) but little is known about the long-term effects of the disease and its treatment on immune function. We report here the assessment of immunity in a group of 62 patients with Hodgkin's disease at presentation and during follow-up for one year from start of treatment; the findings are correlated with method of treatment, splenectomy and progress.

### METHODS

*Peripheral blood lymphocyte counts.*—These were performed at each stage of assessment.

### *Cellular immunity*

*Intradermal skin tests.*—These were performed with 4 or 5 of the following recall antigens: *Candida albicans* antigen 1/100 (Hollister Stiers); mumps virus antigen (Eli Lilly); old tuberculin 1/1000; streptokinase/streptodornase 50 units (Lederle); Trichophyton antigens 1/30 (Hollister Stiers). The skin test sites were each measured at 48 h, and the patient considered immunocompetent if induration of 5 mm or more in one or more test sites was observed.

*Leucocyte-migration inhibition tests.*—The leucocyte-migration inhibition test was modified from the method of Søberg and Bendixen (1967). Separated peripheral leucocytes were allowed to migrate from micro-capillary tubes in wells containing (*a*) purified protein derivative, (*b*) *Candida albicans* extract and (*c*) control medium. Migration areas with antigens in each experiment were statistically compared with control migration areas (Student's *t* test) and significant inhibition of migration was said to have occurred if $P < 0{\cdot}05$. If significant migration inhibition occurred with one or both antigens, the patient was considered immunocompetent in this test.

*Overall assessment of cellular immunity.*—This was obtained by combining the results of skin and leucocyte migration tests, the patient being considered immunocompetent if he

Correspondence to: Dr B. W. Hancock, Department of Medicine, Royal Hospital, Sheffield, S1 3SR.

showed a normal response in either or both tests.

Each patient was also individually assessed in each test of cellular immunity for improvement or deterioration in personal responses.

*Humoral immunity*

Serum immunoglobulin levels were determined by automated immunoprecipitation (Ritchie *et al.*, 1973), the local standard preparation being calibrated in relation to the mass equivalent of the WHO preparation 67/99 (Humphrey and Batty, 1974). The normal adult ranges (g/l serum) in our laboratory are IgG 5·00–16·00, IgA 1·25–4.25, IgM 0·47–1·70.

*Patients*

Sixty-two patients with Hodgkin's disease were assessed at presentation, immediately following radiotherapy or before the third or fourth course of intensive chemotherapy, and either during relapse or after one year's remission.

It is the policy of the Lymphoma Group in Sheffield, U.K., to select patients for laparotomy and splenectomy on the basis of clinical staging and histological type. Those patients with Stage IA–IIA disease of lymphocyte-predominant and nodular-sclerosis histology, and with Stage IIIB–IVA–IVB (all histological types) do not proceed to laparotomy; all other patients do. After pathological staging, patients with Stage I–IIIA disease have radical radiotherapy (mantle, inverted Y or total nodal irradiation); patients with Stage IIIB–IV disease have intensive chemotherapy (modified MOPP, see appendix).

Of the 62 patients, 28 patients underwent splenectomy, and of these, 12 were subsequently treated with chemotherapy, 16 with radiotherapy. Ten of the patients receiving chemotherapy and 9 of those receiving radiotherapy were in complete remission one year after the start of treatment. Two of the chemotherapy and 2 of the radiotherapy group have died (only one with disseminated disease). One of the chemotherapy and 4 of the radiotherapy group relapsed during follow-up, after a period of remission. Of the 34 patients not undergoing splenectomy, 18 had radiotherapy and 16 had chemotherapy. Fourteen of the radiotherapy and 5 of the chemotherapy group were in remission after one year. There were no relapses and only one death (from generalized disease) in the radiotherapy group. In the chemotherapy group, 7 patients died with generalized disease without achieving remission (the non-response group) and 2 patients died of unrelated causes. Two patients relapsed after a period of remission. Three radiotherapy patients were lost to long-term follow-up.

Seven patients (mean age 29·2 ± 9·3 s.e.) were re-assessed during relapse and 38 (mean age 37·9 ± 2·8 s.e.) after one year of remission from the start of therapy. In the latter group 23 patients (9 of whom had had splenectomy) were re-assessed 9–11 months following radiotherapy, and 15 (10 of whom had had splenectomy) before the eighth course of MOPP (*i.e.* after a 3-month period without chemotherapy). In the remission group there were 8 patients with Stage IA disease, 5 with Stage IIA, 2 with Stage IIB, 8 with Stage IIIA, 5 with Stage IIIB, 4 with Stage IVA and 6 with Stage IVB. Histology was lymphocyte-predominant in 2, nodular-sclerosis in 11, mixed-cell in 20 and lymphocyte-depletion in 5 cases. In the relapse group, there were 3 patients with Stage IIA disease, 1 with Stage IIB, 1 with Stage IIIA and 1 with Stage IVA at presentation; the histology was nodular-sclerosis in 4 and mixed-cell in 3 cases. In the non-response group (mean age 47·8 ± 8·7 s.e.) there were 2 patients with Stage IIIB, 1 with Stage IVA and 4 with Stage IVB at presentation; the histology was nodular-sclerosis in 2, mixed-cell in 2 and lymphocyte-depletion in 3 cases.

The results in the remission, relapse and non-response groups were recorded (Table, where the means ± s.e. are shown). To reduce the effects of the variations in the basic levels of each quantitative variable (neutrophils, lymphocytes, immunoglobulins) between the patients, the changes—pre-treatment minus post-treatment on one hand, and pre-treatment minus remission on the other—for each of the variables were considered. As a simple first step, changes within the groups of patients attaining remission, not responding to treatment or subsequently relapsing were analysed separately using Student's $t$ test.

In a further analysis of the data, multiple linear regressions were used in an attempt to relate the changes for each of the quantitative variables to the known factors such as histology, stage, symptoms, splenectomy/no splenectomy, remission/no remission. Little in the way of significant explanatory effects emerged, although this was due not so much to lack of fit of the regression model, as to the large variability in the data.

Patients in remission were then assessed, to relate the relative changes in levels for each of the variables to the two main factors (*i.e.* radiotherapy/chemotherapy and splenectomy/no splenectomy). A two-way analysis of variance with interaction with unequal numbers of observations in the cells (Scheffé, 1960) was used. Inspection of the data revealed that the underlying assumption of normality seemed reasonable.

### RESULTS

(a) *Patients in remission, in relapse or not responding to treatment* (see Table)

In patients achieving remission, neutrophil and lymphocyte counts fell significantly with treatment (both $P < 0{\cdot}001$) before recovering significantly in remission ($P < 0{\cdot}01$ and $< 0{\cdot}001$ respectively). Serum IgG, IgA and IgM levels all fell significantly with treatment ($P < 0{\cdot}05$, $< 0{\cdot}005$ and $< 0{\cdot}01$ respectively); the IgA and IgM levels fell further in remission ($P < 0{\cdot}05$ and $< 0{\cdot}001$ respectively). Minimal depression of overall and skin-test immunocompetence, and improvement in immunocompetence as measured by leucocyte migration inhibition were seen with therapy; the results in remission were similar to those at presentation.

In patients in relapse, leucocyte and immunoglobulin levels showed similar tendencies to those seen in the remission group. Lymphopenia ($< 1{\cdot}0 \times 10^9$/l) was seen in 4/7 patients in relapse and only 2/7 showed normal cellular immunocompetence at this stage.

In assessments of patients failing to respond to treatment, results (before and after therapy) were generally more depressed than in the other groups. Three of the 7 patients in this group had normal immunity at presentation; after treatment only 2 were immunocompetent.

(b) *Patients attaining remission; analysis of changes according to splenectomy and therapy status*

*Leucocyte counts* (Fig. 1).—The drop in neutrophil and lymphocyte counts after treatment was significantly larger ($P < 0{\cdot}05$ and $< 0{\cdot}01$ respectively) in non-splenectomized patients. The change from pre-treatment to final (remission) counts was significantly different ($P < 0{\cdot}025$) only for neutrophil counts, final levels being relatively higher in splenectomized patients.

FIG. 1.—Mean changes in leucocyte counts from before treatment to following treatment (post) and to remission (final) assessments in groups of patients attaining remission. *Key*: RT, Radiotherapy; S, Splenectomy; CT, Chemotherapy; NS, No splenectomy.

*Immunoglobulins* (Fig. 2).—Patients having splenectomy and cytotoxic chemotherapy showed significantly ($P < 0{\cdot}01$) greater falls in IgM and IgG levels than any of the other groups after treatment. In remission, serum IgM levels in splenectomized chemotherapy patients remained low, but the levels in the other groups also fell markedly. From post-treatment to final assessments, the change in levels in non-splenectomized patients was significantly greater than in splenectomized patients ($P < 0{\cdot}05$). The changes in IgG levels from post-treatment to final values were significantly different ($P < 0{\cdot}05$) between chemotherapy and radiotherapy

TABLE.—*Immune Status of Patients with Hodgkin's Disease (Results of Follow-up Assessments in Remission, Relapse and Non-response Groups)*

| | Remission (38 patients) | | | Relapse (7 patients) | | | Non-response (7 patients) | |
|---|---|---|---|---|---|---|---|---|
| | Pre-treatment | Post-treatment | Remission | Pre-treatment | Post-treatment | Relapse | Pre-treatment | Post-treatment |
| Neutrophil count ($\times 10^9$/l mean ±s.e.) | 5·47±0·44 | 3·65±0·33 | 4·67±0·31 | 7·57±1·64 | 4·39±0·82 | 6·14±1·36 | 6·47±1·59 | 3·20±0·37 |
| Lymphocyte count ($\times 10^9$/l mean ±s.e.) | 1·70±0·09 | 1·03±0·15 | 1·72±0·12 | 1·51±0·13 | 0·68±0·09 | 1·09±0·25 | 1·18±0·09 | 1·00±0·18 |
| Immunoglobulins (g/l mean ±s.e.) | | | | | | | | |
| IgG | 12·13±0·42 | 10·77±0·62 | 11·24±0·56 | 13·23±1·70 | 14·28±2·03 | 11·84±1·68 | 9·44±1·56 | 6·27±0·94 |
| IgA | 2·83±0·28 | 2·16±0·20 | 1·63±0·15 | 2·69±0·67 | 2·44±0·37 | 1·42±0·36 | 2·44±0·49 | 1·83±0·46 |
| IgM | 1·21±0·12 | 0·91±0·09 | 0·55±0·05 | 1·06±0·22 | 0·95±0·20 | 0·54±0·12 | 1·10±0·19 | 0·66±0·23 |
| Skin test responses (% immuno-competent) | 79 | 66 | 81 | 71 | 14 | 14 | 43 | 0 |
| Leucocyte migration responses (% immunocompetent) | 42 | 55 | 34 | 14 | 43 | 28 | 14 | 28 |
| Overall cellular immuno-competence (% competent in skin and/or leucocyte migration responses) | 84 | 76 | 87 | 71 | 57 | 28 | 43 | 28 |

FIG. 2.—Mean changes in serum immunoglobulin levels from before treatment to following treatment (post) and to remission (final) assessments in groups of patients attaining remission. *Key*: RT, Radiotherapy; S, Splenectomy; CT, Chemotherapy; NS, No splenectomy.

groups, the former groups showing increasing and the latter groups decreasing levels.

*Cellular immunity*.—The changes in cellular immunity between groups mirrored those of the remission groups as a whole, although skin-test and overall immunocompetence were more depressed by chemotherapy than by radiotherapy. Splenectomy did not obviously influence cellular immune function after treatment or in remission.

(c) *Correlations with infection*.—Infections are a recognized feature of Hodgkin's disease, particularly patients with advanced disease and undergoing immunosuppressive treatment (Casazza, Duvall and Carbone, 1966).

The cause of death in patients not responding to treatment was invariably bronchopneumonia. In these patients there was marked deterioration in cellular immunity following treatment; levels in all immunoglobulin classes fell more than in the other groups (Table).

Three patients in the splenectomy group died of fulminating septicaemia (Hancock *et al.*, 1976*a*). Pre-terminal immune assessment showed deteriorating cellular immunity and inappropriately low IgM levels.

Three patients (2 having undergone splenectomy) developed herpes zoster infection during the follow-up period. In 2 this was associated with deteriorating cellular immunity and preceded clinical relapse. In the third patient, infection followed radical radiotherapy, when cellular immunity, previously normal, was depressed; this patient achieved, and is still in, remission and cellular immunity is again normal.

Two patients (both splenectomized) developed varicella infection, one during total nodal irradiation and the other during intensive chemotherapy. In both cases, temporary depression of cellular immunity was noted with treatment, and in one case varicella was followed by severe oral candidiasis; both patients achieved, and are still in, remission with normal immunity.

No bizarre opportunistic infections were seen but one totally anergic patient with Stage IVB disease (lymphocyte-depleted histology) responding only partially to chemotherapy, developed renal candidiasis as a terminal feature.

The list of infections discussed above is not exhaustive, as trivial non-specific respiratory and mucosal infections were not documented.

### DISCUSSION

Variable changes in the immunological status of patients with Hodgkin's disease in the period following radical radiotherapy or intensive chemotherapy have been reported. Both radiotherapy (Gross, Manfredi and Protos, 1973; Chee, Illberg and Rickinson, 1974; Raben *et al.*, 1976) and chemotherapy (review—Harris *et al.*, 1976) may depress immunity, but the improvement in the patients' general condition following remission of the malignancy may result in normal or improved immune function (Sokal and Primikirios, 1961; Young *et al.*, 1972). In patients with Hodgkin's disease studied 5 years after completing radical radiotherapy, no gross defects in immunity were found (Kun and Johnson, 1975). Short intensive courses of chemotherapy, whilst initially suppressing humoral and cellular immunity, may be followed by "rebound-overshoot" recovery (Serrou, Dubois and Silva, 1974; Harris *et al.*, 1976) and even after prolonged continuous chemotherapy, immunity may be only slightly depressed or may even recover to normal (Chang, Stutzman and Sokal, 1975). Cessation of long-term therapy is followed by rapid recovery of any depression of immune function (Borella, Green and Webster, 1972).

Such observations may indicate differences in methods of assessment of immunity and the variable immunosuppressive effects of cytotoxic drugs and therapeutic regimes being used.

In a preliminary report (Hancock *et al.*, 1976*b*) of our follow-up results, we found that, regardless of whether splenectomy had been performed, overall cellular immunity deteriorated with intensive treatment, particularly chemotherapy. Skin reactivity deteriorated, especially in chemotherapy patients, even though leucocyte migration reactivity improved marginally. Serum immunoglobulins, and particularly IgM levels, fell during intensive therapy in splenectomized patients. Three patients in the latter group died of fulminating septicaemia during or shortly after treatment (Hancock *et al.*, 1976*a*) and it may be that IgM immunoparesis was a contributing feature in the genesis of their infections.

The present study confirms many of our previous findings, but further analysis shows that early falls in serum IgG and IgM classes are seen with intensive chemotherapy in splenectomized patients.

In the present follow-up study, 6 patients (4 splenectomy, 2 non-splenectomy) developed viral or fungal infections during or following intensive treatment. All had shown marked deterioration of cellular immunity, and two subsequently relapsed. No further major bacterial infections have been seen, except in those dying with disseminated disease, when the terminal event was invariably bronchopneumonia.

One year after the start of therapy, patients in remission showed no major differences in cellular immunity from their initial assessments, though individual patients showed conversion from non-reactivity to reactivity and *vice versa.* Splenectomy or mode of therapy did not ultimately affect the assessment of cellular immunity in remission. At remission, serum immunoglobulin levels were generally lower than after treatment, with the exception that IgG levels were recovering in patients having maintenance chemotherapy. The fall in IgA levels was much less in non-splenectomized patients having radiotherapy. It is of importance that the early major differences in the fall in IgM levels between the splenectomized patients having chemotherapy and all other groups was considerably reduced by the time of remission.

The trends in the groups of patients not responding to treatment or subsequently relapsing were similar to those attaining remission, but there were some obvious differences in cellular immunity. Lymphopenia was common in patients in relapse, and in the relapse group as a whole cellular immunity was markedly depressed, probably as a marker of active disease. Cellular immunity in these patients, and strikingly in the non-response cases, was worse than with the remission group at presentation and after treatment. In the case of the non-response group, these findings may be related to the high incidence of generalized disease and lymphocyte-depleted histology at presentation.

The relative depression of humoral immunity expected with advanced disease (Aisenberg and Leskowitz, 1963) was not seen, with the exception that in the non-response group low initial and post-treatment serum IgG levels were demonstrated.

It has been suggested that splenectomized patients are better able to tolerate radiotherapy (Saltzman and Kaplan, 1971) and cytotoxic drugs (Pannetière and Coltman, 1973). Certainly our study confirms a possible "protective" effect on peripheral leucocyte counts in splenectomized patients, counts in these patients remaining higher than in those not having splenectomy.

In conclusion it seems that regular assessment of immune status in patients with Hodgkin's disease may prove a marker of disease activity. Relapse or non-response seems to be associated with deteriorating, and remission with improving, cellular immunity. The importance of the falling immunoglobulin levels in patients in remission at one year, and particularly the early fall in IgM and IgG in splenectomized patients receiving chemotherapy, is uncertain, but the possible role of IgM immunoparesis as a predisposing factor in the development systemic sepsis must be carefully assessed by continued observation of these patients.

We are grateful to the consultant medical staff of Weston Park Hospital whose patients have been studied; and to the Cancer Research Campaign (Yorkshire Branch) for financial assistance.

### APPENDIX

*Intensive cyclical chemotherapy*

*Modified MOPP regime*

| | |
|---|---|
| Mustine 6 mg/m$^2$ i.v.<br>Vincristine (Oncovin) 1·4 mg/m$^2$ i.v. | Days 1 and 8 |
| Oral procarbazine 100 mg/m$^2$<br>Oral prednisolone 40 mg/day | Days 1–14 |

Six courses beginning at 28-day intervals. Then 4 further courses at 3-monthly intervals.

### REFERENCES

AISENBERG, A. C. & LESKOWITZ, S. (1963) Antibody Formation in Hodgkin's Disease. *N. Engl. J. Med.*, **268**, 1269.

BORELLA, L., GREEN, A. A. & WEBSTER, R. G. (1972) Immunologic Rebound after Cessation of Long-term Chemotherapy in Acute Leukaemia. *Blood*, **40**, 42.

CASAZZA, A. R., DUVALL, C. P. & CARBONE, P. P. (1966) Summary of Infectious Complications Occurring in Patients with Hodgkin's Disease. *Cancer Res.*, **26**, 1290.

CHANG, T. C., STUTZMAN, L. & SOKAL, J. E. (1975) Correlation of Delayed Hypersensitivity Responses with Chemotherapeutic Results in Advanced Hodgkin's Disease. *Cancer, N.Y.*, **36**, 950.

CHEE, C. A., ILLBERG, P. L. T. & RICKINSON, A. B. (1974) Depression of Lymphocyte Replicating Ability in Radiotherapy Patients. *Br. J. Radiol.*, **47**, 37.

GROSS, L., MANFREDI, D. L. & PROTOS, A. A. (1973) The Effect of Cobalt 60 Irradiation upon Cell Mediated Immunity. *Radiology*, **106**, 653.

HANCOCK, B. W., BRUCE, L., MILFORD WARD, A. & RICHMOND, J. (1976*a*) Changes in Immune Status in Patients undergoing Splenectomy for the Staging of Hodgkin's Disease. *Br. med. J.*, i, 313.

HANCOCK, B. W., BRUCE, L., MILFORD WARD, A. & RICHMOND, J. (1976*b*) Effects of Therapy and Splenectomy on Immunity in Malignant Lymphoma (Abstract). *Clin. Sci. mol. Med.*, **51**, 24p.

HANCOCK, B. W., BRUCE, L., SUGDEN, P., MILFORD WARD, A. & RICHMOND, J. (1977) Immune Status in Untreated Patients with Lymphoreticular Malignancy: a Multifactorial Study. *Clin. Oncol.*, **3**, 57.

HARRIS, J., SENGAR, D., STEWART, T. & HYSLOP, D. (1976) The Effect of Immunosuppressive Chemotherapy on Immune Function in Patients with Malignant Disease. *Cancer, N.Y.*, **37**, 1058.

HUMPHREY, J. H. & BATTY, I. (1974) International

Reference Preparation for Human Serum IgG, IgA and IgM. *J. Immunol.*, **113**, 428.

KUN, L. E. & JOHNSON, R. E. (1975) The Haematologic and Immunologic Status in Hodgkin's Disease 5 Years after Radical Radiotherapy. *Cancer, N.Y.*, **36**, 1912.

PANNETIÈRE, F. & COLTMAN, C. A. (1973) Splenectomy Effects on Chemotherapy in Hodgkin's Disease. *Arch. intern. Med.*, **131**, 362.

RABEN, M., WALACH, N., GALILI, U. & SCHLESINGER, M. (1976) The Effect of Radiation Therapy on Lymphocyte Sub-populations in Cancer Patients. *Cancer, N.Y.*, **37**, 1417.

RITCHIE, R. F., ALPER, C. A., GRAVES, J., PEARSON, N. & LARSON, C. (1973) Automated Quantitation of Proteins in Serum and Other Biological Fluids. *Am. J. clin. Path.*, **59**, 151.

SALTZMAN, J. R. & KAPLAN, H. S. (1971) Effect of Prior Splenectomy on Haematologic Tolerance during Total Lymphoid Radiotherapy in Patients with Hodgkin's Disease. *Cancer, N.Y.*, **27**, 471.

SCHEFFÉ, H. (1960) *Analysis of Variance*. New York: Wiley. p. 112.

SERROU, B., DUBOIS, J. B. & SILVA, R. (1974) The Immunological Overshoot Phenomenon (I.O.P.) in Chemotherapy of Solid Tumours. *Proc. Am. Assoc. Cancer Res.*, **15**, 125.

SOBERG, M. & BENDIXEN, G. (1967) Human Lymphocyte Migration as a Parameter of Hypersensitivity. *Acta med. scand.*, **181**, 247.

SOKAL, J. & PRIMIKIRIOS, N. (1961) Delayed Skin Test Response in Hodgkin's Disease and Lymphosarcoma. Effect of Disease Activity. *Cancer, N.Y.*, **14**, 597.

YOUNG, R. C., CORDER, M. P., HAYNES, H. A. & DEVITA, V. T. (1972) Delayed Hypersensitivity in Hodgkin's Disease; a Study of 103 Untreated Patients. *Am. J. Med.*, **52**, 63.


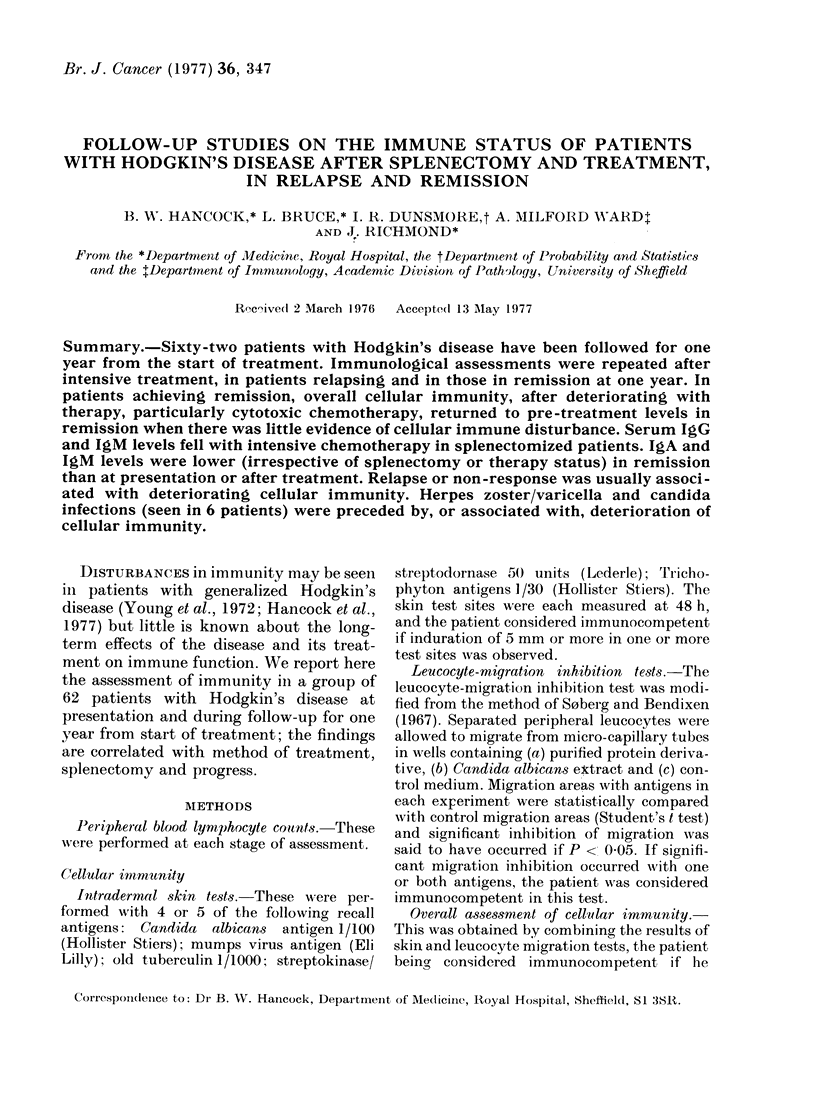

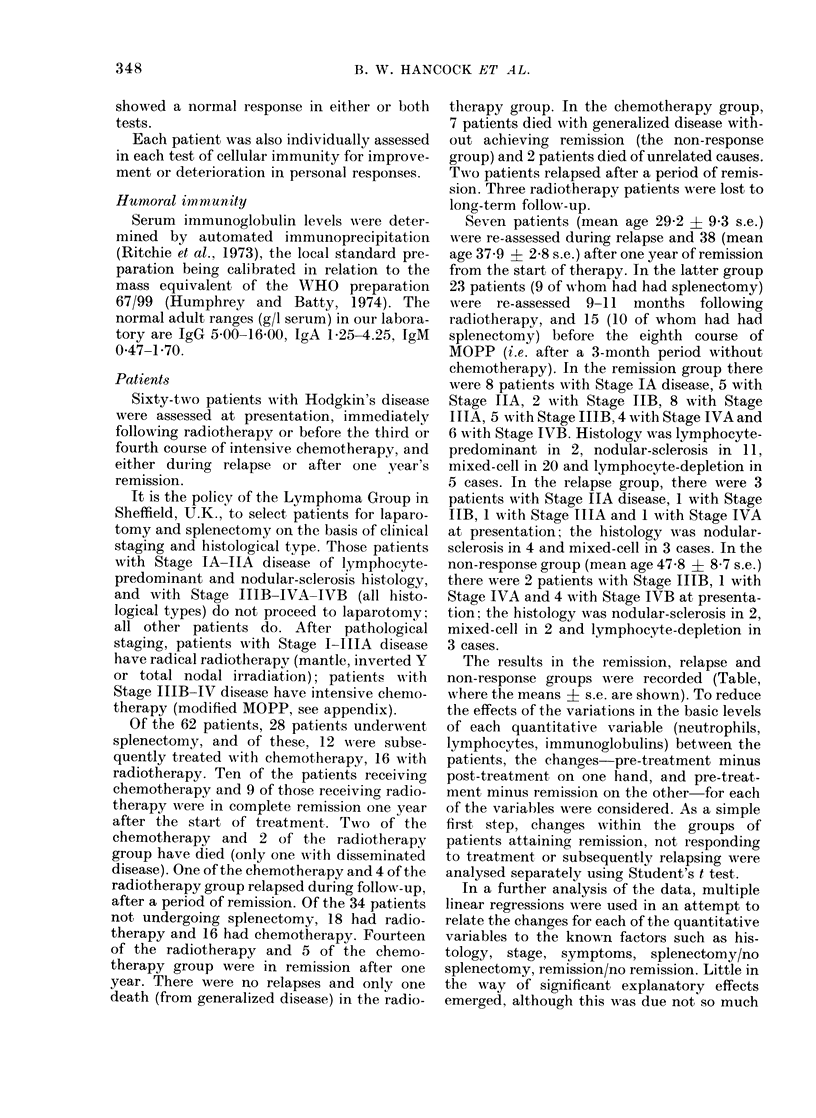

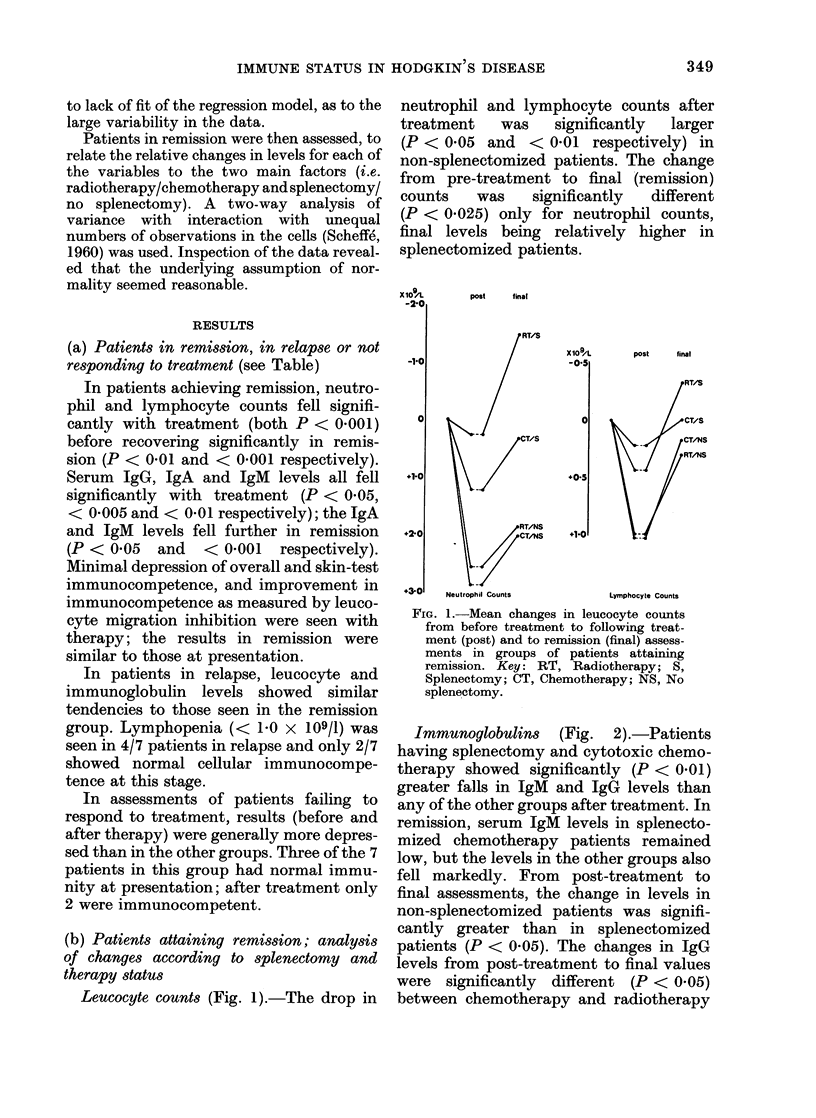

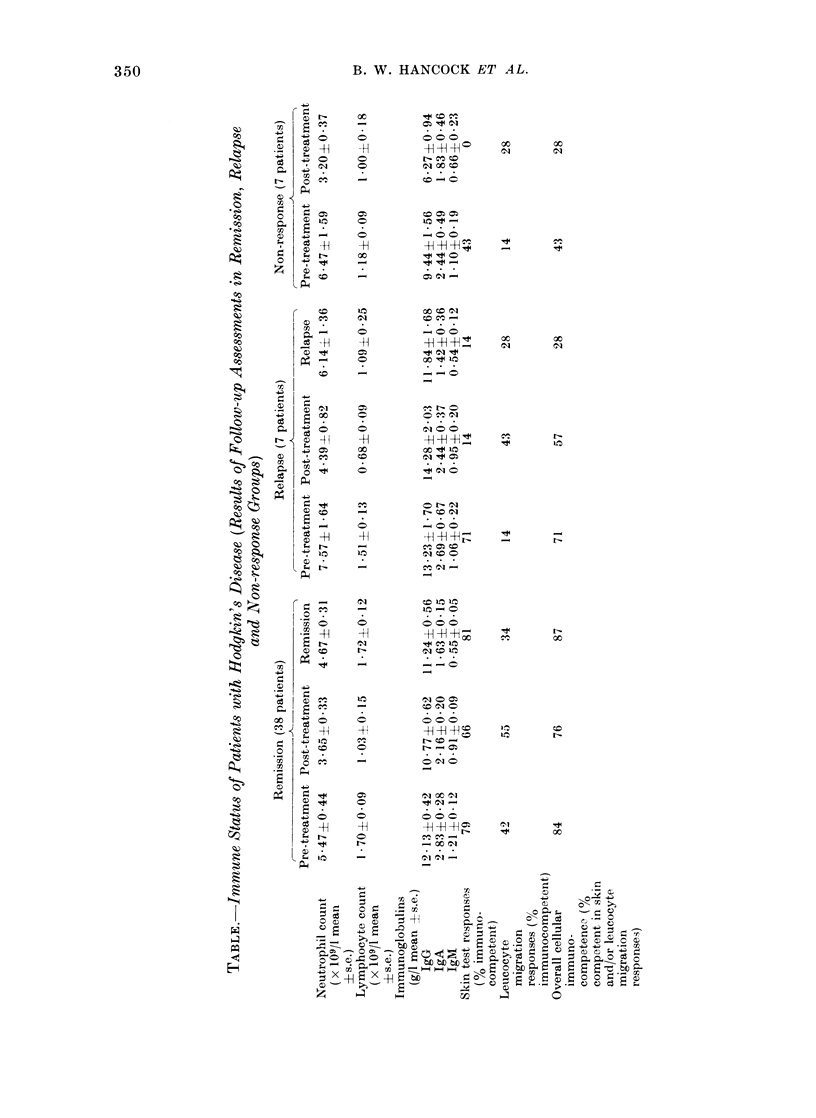

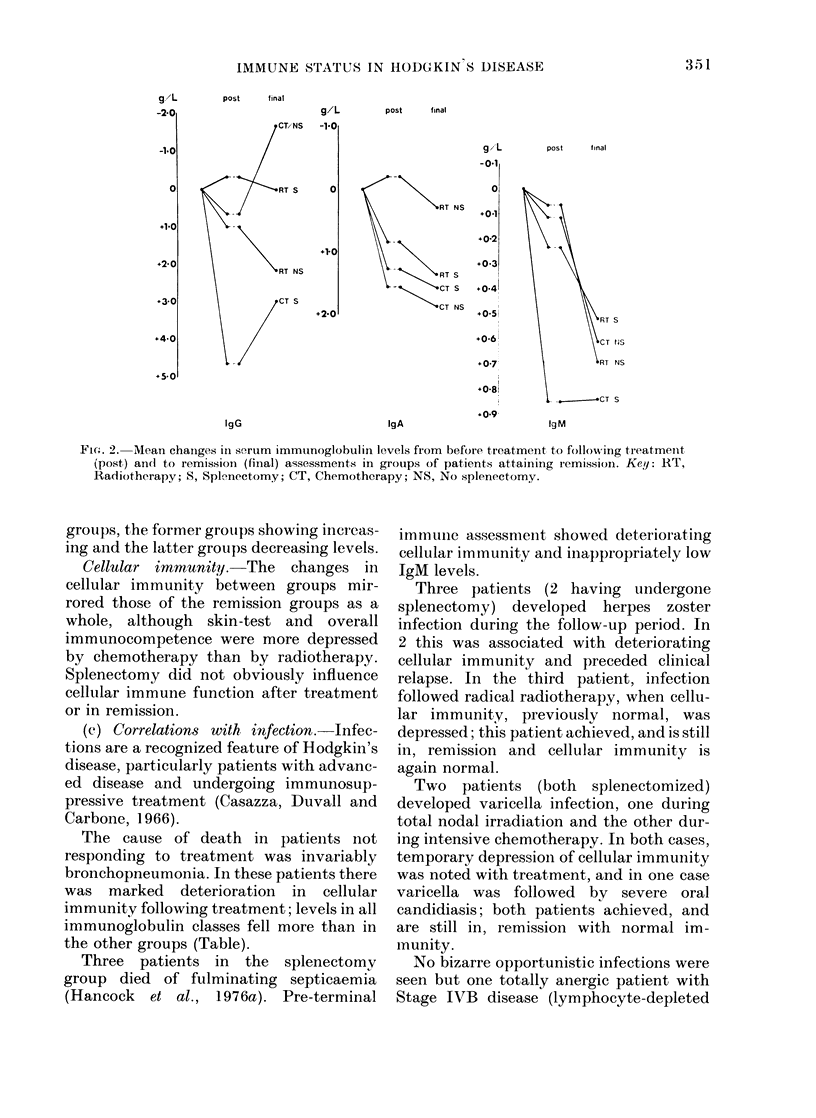

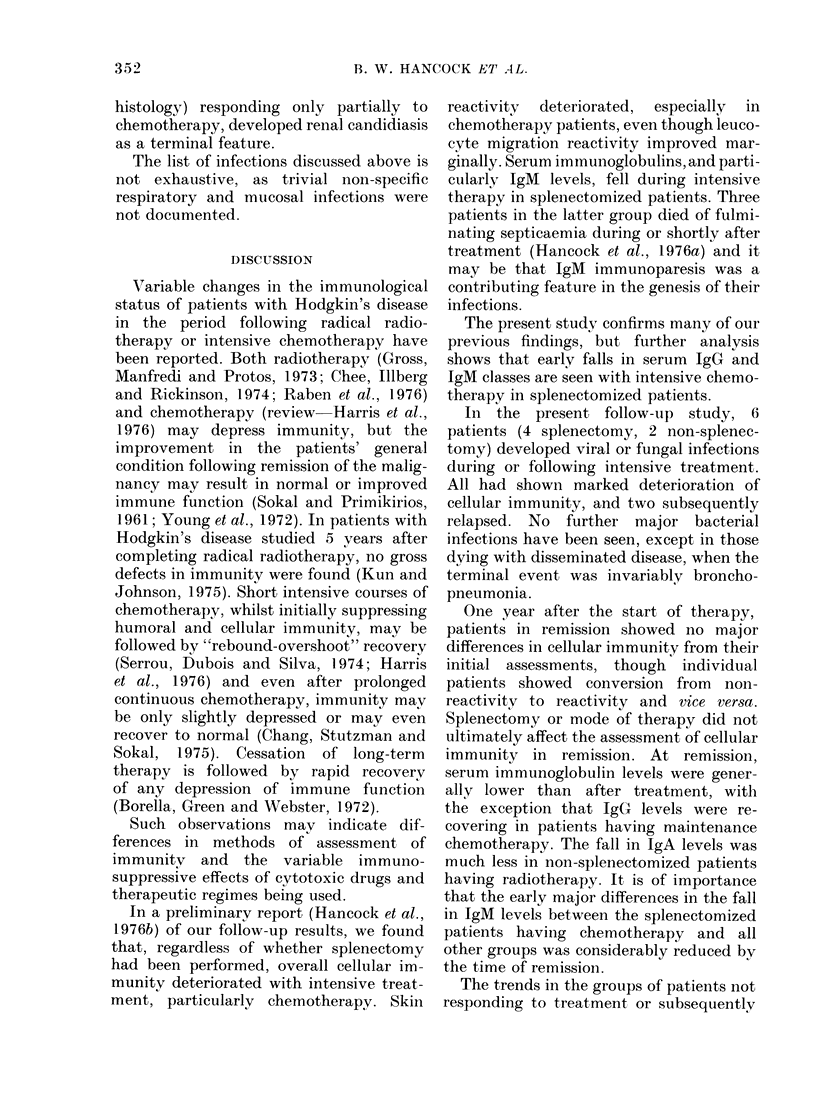

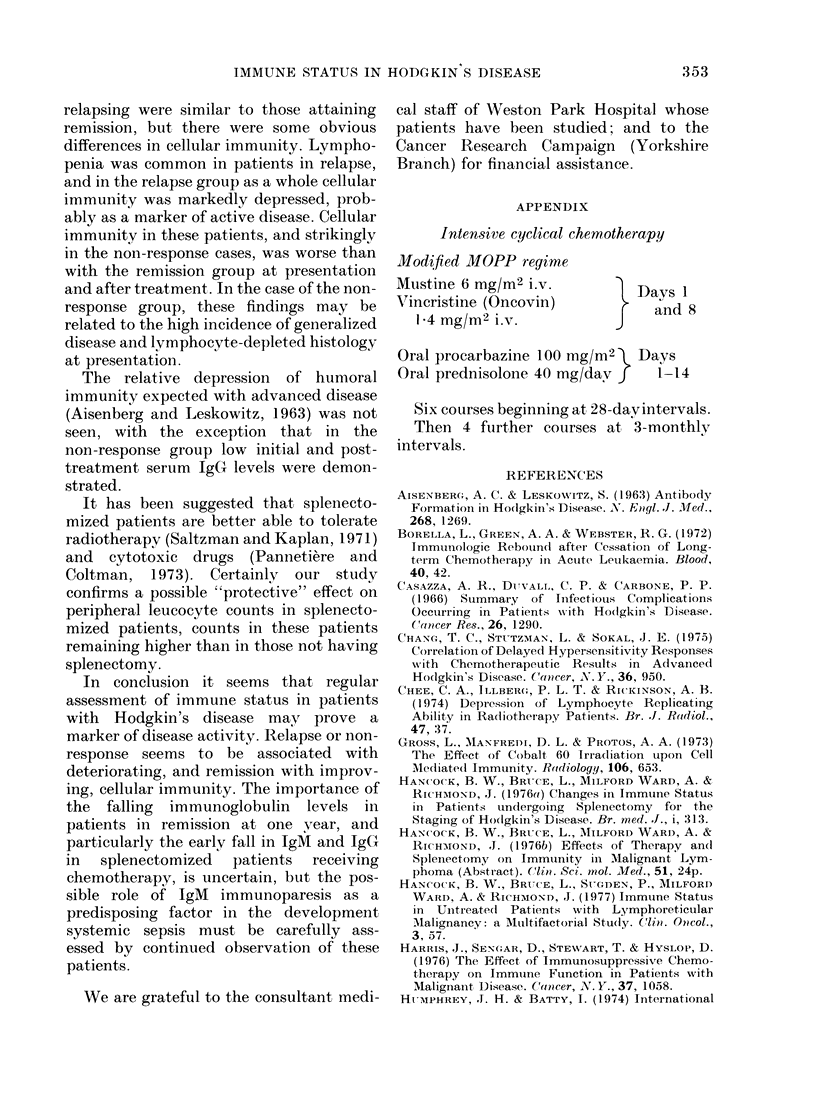

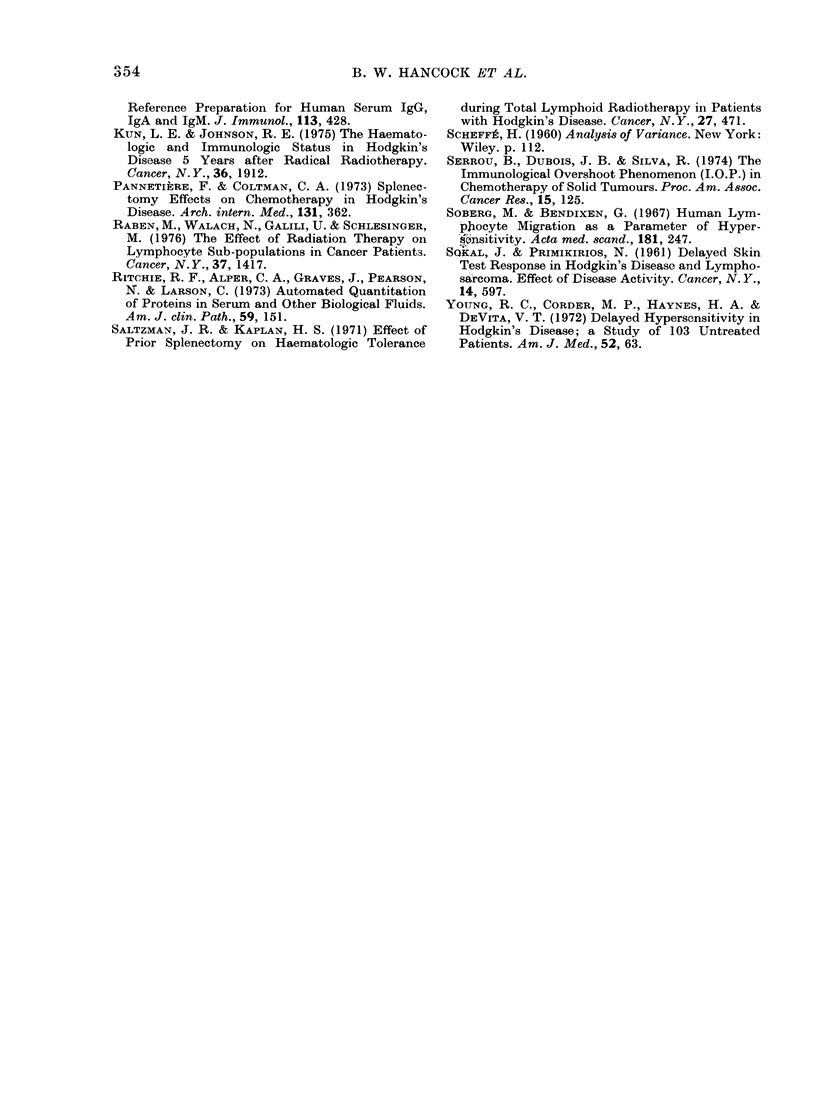


## References

[OCR_00801] Borella L., Green A. A., Webster R. G. (1972). Immunologic rebound after cessation of long-term chemotherapy in acute leukemia.. Blood.

[OCR_00815] Chang T. C., Stutzman L., Sokal J. E. (1975). Correlation of delayed hypersensitivity responses with chemotherapeutic results in advanced Hodgkin's disease.. Cancer.

[OCR_00825] Gross L., Manfredi O. L., Protos A. A. (1973). Effect of cobalt-60 irradiation upon cell-mediated immunity.. Radiology.

[OCR_00848] Harris J., Sengar D., Stewart T., Hyslop D. (1976). The effect of immunosuppressive chemotherapy on immune function in patients with malignant disease.. Cancer.

[OCR_00861] Kun L. E., Johnson R. E. (1975). Hematologic and immunologic status in Hodgkin's disease 5 years after radical radiotherapy.. Cancer.

[OCR_00867] Panettiere F., Coltman C. A. (1973). Splenectomy effects on chemotherapy in Hodgkin's disease.. Arch Intern Med.

[OCR_00872] Raben M., Walach N., Galili U., Schlesinger M. (1976). The effect of radiation therapy on lymphocyte subpopulations in cancer patients.. Cancer.

[OCR_00878] Ritchie R. F., Alper C. A., Graves J., Pearson N., Larson C. (1973). Automated quantitation of proteins in serum and other biologic fluids.. Am J Clin Pathol.

[OCR_00884] Salzman J. R., Kaplan H. S. (1971). Effect of prior splenectomy on hematologic tolerance during total lymphoid radiotherapy of patients with Hodgkin's disease.. Cancer.

[OCR_00901] Soborg M., Bendixen G. (1967). Human lymphocyte migration as a parameter of hypersensitivity.. Acta Med Scand.

[OCR_00912] Young R. C., Corder M. P., Haynes H. A., DeVita V. T. (1972). Delayed hypersensitivity in Hodgkin's disease. A study of 103 untreated patients.. Am J Med.

